# Oral manifestations associated with neutropenia in Syrian patients diagnosed with hematological malignancies and undergoing chemotherapy: A cross-sectional study

**DOI:** 10.1097/MD.0000000000036780

**Published:** 2024-01-12

**Authors:** Fatima AlZahraa Al Beesh, Nafiza Martini, Siham Suleiman, Abeer Aljoujou

**Affiliations:** a University of Damascus, Faculty of Dentistry, Department of Oral Medicine, Damascus, Syrian Arab Republic; b University of Damascus, Faculty of Medicine, Damascus, Syrian Arab Republic; c Stemosis for Scientific Research, Damascus, Syrian Arab Republic; d University of Damascus, Faculty of Medicine, Department of Hematology-Oncology, Damascus, Syrian Arab Republic.

**Keywords:** chemotherapy induced neutropenia, infections, neutropenia, oral manifestations, oral mucositis, oral ulcers

## Abstract

Neutropenia can be caused by a variety of congenital and acquired factors, with Chemotherapy-induced myelosuppression being the most common cause. Neutropenia significantly affects oral health, leading to the manifestation of oral lesions such as ulcers, fungal and viral infections, and mucositis. This study aims to investigate oral lesions in patients with hematological malignancies who developed neutropenia after chemotherapy. This cross-sectional study included 50 patients with hematological malignancies. The participants were divided into 2 groups: the first group consisted of 25 patients with hematological malignancies who developed chemotherapy-induced neutropenia and the second group consisted of 25 patients with hematological malignancies who did not develop chemotherapy-induced neutropenia. Patients were assigned to one of the groups based on the absolute neutrophil count (ANC). Full oral clinical examination was performed to determine the presence of oral lesions. In the Chemotherapy-Induced Neutropenia group, the most common lesion was ulceration, observed in 12 patients (48%). Fungal infections were the second most common, present in 5 patients (20%), followed by viral infections in 4 patients (15%), and mucositis, which occurred in a single patient (4%). A statistically significant association was found between neutropenia and the presence of oral ulcers (*P* value = .015). In contrast, in the Chemotherapy group, oral changes were less frequent. Fungal infections were the most common, occurring in 4 patients (15%), followed by oral mucositis in 3 patients (12%). Ulceration and viral infections were the least common, each observed in 1 patient (4%). The frequency of various forms of oral ulcers increases with the severity of neutropenia. However, there was no significant increase in other oral lesions in patients with neutropenia.

## 1. Introduction

Neutrophils, polymorphonuclear leukocytes, are the predominant innate immune cells in the human body. They are produced primarily in the bone marrow and serve crucial functions, such as the elimination of bacterial, viral, and fungal pathogens, as well as aiding in acute inflammatory responses, facilitating tissue repair enhancing overall oral health.^[1–4]^ Therefore, any disturbance in neutrophil count can result in adverse oral consequences such as ulcers and infections.^[5]^

Neutropenia, characterized by a decrease in the absolute neutrophils count (ANC) below the normal range,^[6]^ can arise from various factors, including both congenital and acquired conditions, but chemotherapy is the primary cause.^[7]^

It can be classified by severity into: mild (ANC from 1.5 to 1.0 × 10^^^9/L), moderate (ANC from 1.0 to 0.5 × 10^^^9/L), severe (ANC from 0.5 to 0.2 × 10^^^9/L), and very severe (ANC <0.2 × 10^^^9/L).^[8]^

Chemotherapy is the standard treatment for hematological malignancies.^[9]^ The primary goal of chemotherapy is to target and inhibit the proliferation of rapidly dividing cells. However, they lack the ability to differentiate between cancer and healthy cells, such as those found in the bone marrow and oral mucosa. Therefore, oral manifestations and side effects may occur after chemotherapy.^[10–12]^

Chemotherapy and neutropenia can lead to various oral manifestations including; oral ulcers, infections, oral mucositis, and periodontal disease. These conditions are among the potential oral consequences of chemotherapy and their effects on neutrophil levels.^[12–15]^

This study aimed to investigate the occurrence of oral lesions in patients with hematological malignancies who developed neutropenia after chemotherapy.

## 2. Methods

### 2.1. Participants and study design

This cross-sectional study was conducted between October 2021 and January 2023 at various medical institutions, including the Hematology Oncology Department of Al-Bairouni University Hospital, Al-Assad University Hospital, and the Department of Oral Medicine, Faculty of Dentistry, Damascus University. The primary objective of this study was to perform clinical oral examinations in patients diagnosed with haematological malignancies who underwent chemotherapy throughout the study period. The study total sample comprised 50 patients who were categorized into 2 distinct groups based on their absolute neutrophil counts:

1. The first group, referred to as the Chemotherapy-Induced Neutropenia group (CIN) group, consisted of 25 patients who developed neutropenia as a consequence of chemotherapy.
2. The second group, referred to as the chemotherapy group, comprised 25 patients who did not experience neutropenia after chemotherapy.
1. The inclusion criteria for the study were as follows:
CIN group:
  - Patients diagnosed with hematological malignancy.
  - Patients who received at least one cycle of chemotherapy.
  - Patients who developed neutropenia following chemotherapy, with an ANC <1.5 × 10^^^9/L
Chemotherapy group:
  - Patients diagnosed with hematological malignancy.
  - The patient received at least one cycle of chemotherapy.
  - The patient did not present with chemotherapy-induced neutropenia, and had an ANC >1.5 × 10^^^9/L.
2. The exclusion criteria for the study were as follows:
  - Patients who received radiation therapy as part of their treatment.
  - Patients diagnosed with oral cancer.
  - Patients diagnosed with non-hematological malignancy.
  - Patient diagnosed with diabetes mellitus.

### 2.2. Clinical oral examination

Personal data were collected through thorough examination of each patient medical records, assessment of their overall condition, and review of recent blood test results. A clinical examination was conducted to identify specific oral lesions based on their clinical appearance, such as ulcers, fungal and viral infections, and oral mucositis.

### 2.3. Sample size

The sample size for the present study was determined using G*Power 3.1.9.2 software, with a significance level set at 0.05, confidence level at 0.95, and a size effect of 0.95, based on a previous similar study conducted by Park in 2011.^[16]^

### 2.4. Statistical analysis

Kendall Tau test was used to examine the statistical relationship between the occurrence of neutropenia and the development of oral lesions, and Cramer V-test was used to investigate the relationship between chemotherapy and the development of oral lesions. Results were considered significant if *P* value < .05.

### 2.5. Ethics

The Scientific Research Committee of the Faculty of Dentistry of Damascus University obtained the necessary regulatory approvals to conduct the research, and the study was registered with number 154 on 27/09/2021, written informed consent after receiving a comprehensive explanation of the study objectives, procedures, and considerations regarding patient privacy.

This study is registered in the ISRCTN with the number 17585958.

## 3. Results

**Figure 1.**
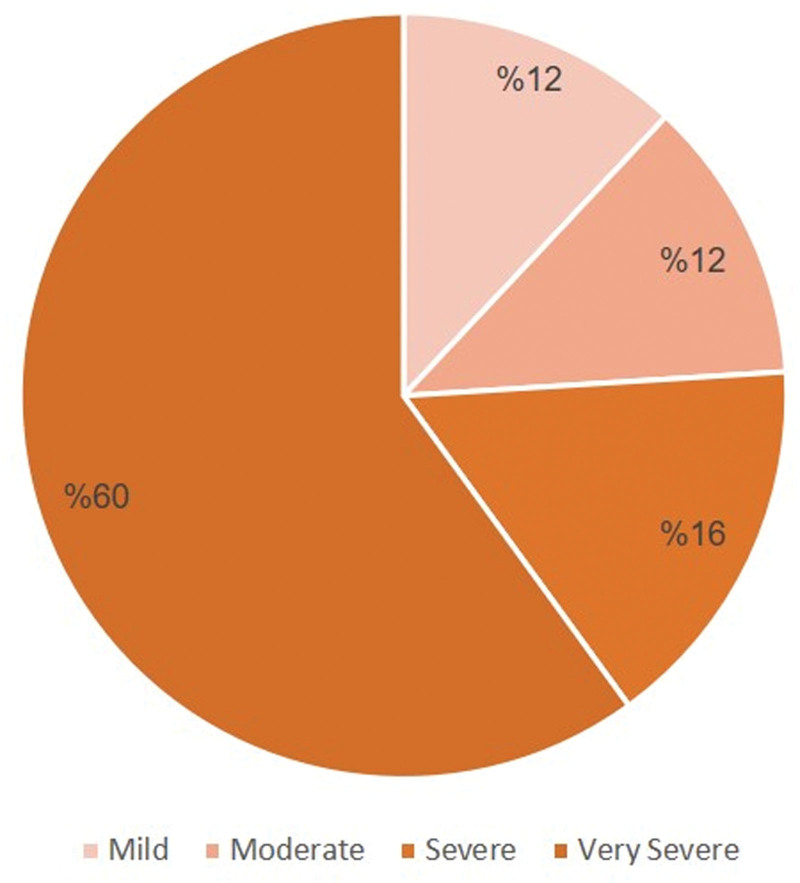
Shows the severity of neutropenia within the chemotherapy-induced neutropenia group.

A total of 50 patients (29 males, 21 females) participated in this study. There were 25 patients in the CIN group (14 males, 11 females) with a mean age of 34.64 years. There were 25 patients in the chemotherapy group (15 males, 10 females) with a mean age of 39 years. The neutropenic group consisted of 3 patients (12%) with mild neutropenia, 3 patients (12%) with moderate neutropenia, 4 patients (16%) with severe neutropenia, and 15 patients (60%) with very severe neutropenia (Fig. 1).

### 3.1. Oral findings

**Figure 2.**
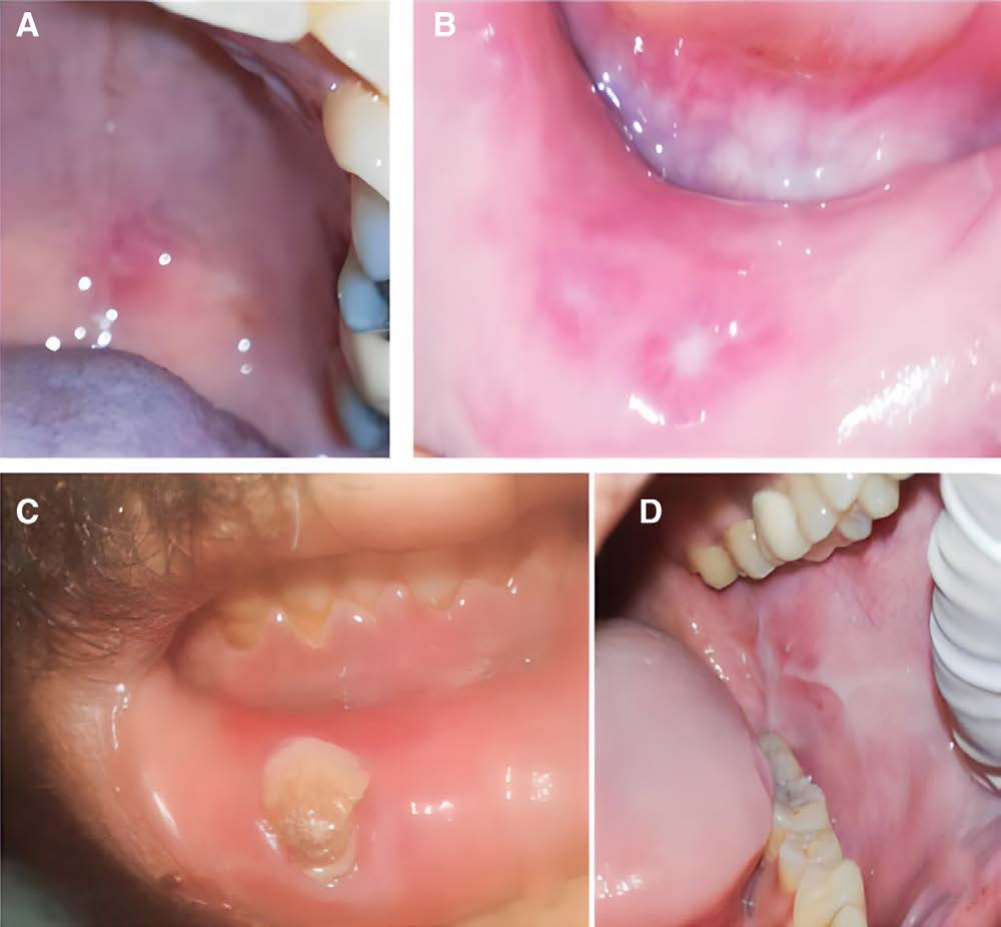
Shows several oral ulcers. Specifically, (A to C) show ulcers in neutropenic patients, (A) and (B) represent aphthous ulcers, while (C) depict the typical appearance of a neutropenic ulcer. (D) shows aphthous ulcer observed in a leukemic patient who was undergoing chemotherapy but did not develop neutropenia.

**Figure 3.**
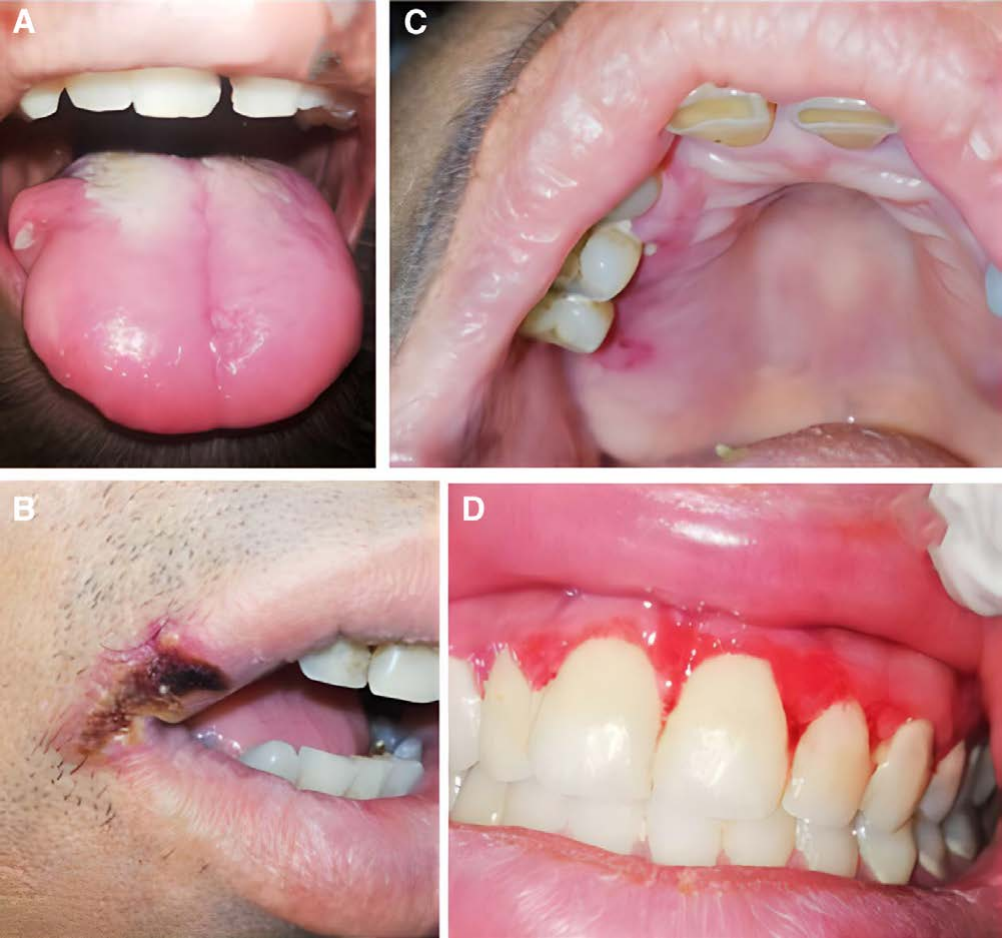
Shows infections and oral mucositis. (A) shows pseudomembranous candidiasis on the tongue surface in a neutropenic patient, (B) shows an infection caused by HSV-1 in a neutropenic patient, which presents as recurrent herpes labialis, (C) displays oral mucositis in a neutropenic patient and is characterized by the presence of localized erythematous lesions, and (D) displays chemotherapy-induced oral mucositis and is characterized by the presence of diffuse erythematous lesions. HSV = herpes simplex virus.

**Figure 4.**
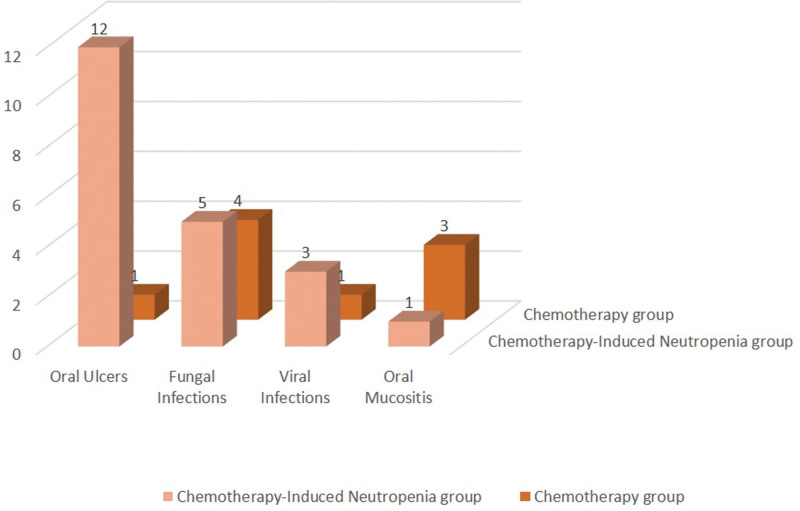
Shows the frequency of oral lesions observed in the study sample.

Significant difference was observed in the incidence rate of oral ulcers between the 2 groups. In the CIN group, almost half of the patients (48%) had different types of oral ulcers (Fig. 2A–C) while only 1 patient (4%) in the chemotherapy group exhibited oral ulcers (Fig. 2D). These findings suggest that the occurrence of oral ulcers increases as neutrophil count decreases. However, no significant association was detected between neutropenia and other studied oral lesions, as the incidence rates of fungal and viral infections were similar in both groups; fungal infections were identified in 5 patients (20%) in the CIN group whereas 4 patients (16%) in the chemotherapy group had such infections (Fig. 3A). and viral infections were observed in 3 patients (12%) in the CIN group (Fig. 3B) while viral infections were detected in only 1 patient (4%) in the chemotherapy group. Oral mucositis was observed in only 1 patient (4%) in the CIN group (Fig. 3C) and in 3 patients (12%) in the chemotherapy group (Fig. 3D). These results suggest that chemotherapy may not considerably influence the development of the studied oral lesions. Comprehensive details of these findings are presented in Figure 4 and Table 1.

**Table 1:** Provides the results of the statistical tests conducted to examine the relationship between the occurrence of oral lesions and the study sample.

		Chemotherapy-induced neutropenia group					*P* value^a^	Chemotherapy Group	*P* value^b^
		Mild	Moderate	Severe	Very severe	Total			
Oral Ulcers	Yes	3	1	3	5	12	.01*	1	.66
	No	0	24	1	10	13		24	
Fungal Infection	Yes	0	0	2	3	5	.40	4	.33
	No	3	3	2	12	20		21	
Viral Infection	Yes	0	0	1	2	3	.32	1	.85
	No	3	3	3	13	22		24	
Oral Mucositis	Yes	0	0	0	1	1	.40	3	.58
	No	3	3	4	14	24		22	

CIN = chemotherapy-induced neutropenia.

a Kendall Tau test,

b Cramer V test,

* statistically significant.

## 4. Discussion

Chemotherapy, as the primary treatment for malignancy, can lead to the development of oral lesions due to various factors. The rapid rate of cell division in the oral mucosa, the presence of diverse oral flora, and the vulnerability of tissues to injury during normal processes make the oral mucosa particularly susceptible to the effects of chemotherapy.^[10,17]^ Accordingly, chemotherapy can directly damage the oral tissues by impairing mucosal regeneration, leading to mucosal atrophy, inflammation, and ulceration.^[11]^ It can also indirectly impact the oral mucosa by causing myelosuppression, which results in neutropenia; Neutropenia is a common complication arising from myelosuppression.^[8,18,19]^ And thus this condition plays a significant role in the development of oral symptoms, particularly ulcers which are often the initial indication of neutropenia, serving as a significant sign of underlying neutropenic condition.^[6,9,20]^ These ulcers typically appear as multiple, irregularly shaped, deep, and painful lesions with a yellow pseudo-fibrinous membrane. They may or may not show peripheral inflammation or erythematous halos. The necrotic tissue of these ulcers may emit an unpleasant odor, but they typically exhibit significant improvement with an increase in the ANC.^[13,20,21]^ Therefore, when evaluating the occurrence of oral ulcers in patients with malignancies, it is essential to consider multiple factors including neutropenia, direct drug toxicity, and infection.^[21]^ To our knowledge, no direct comparisons have been made between CIN and chemotherapy alone in the development of oral ulcers. However, our study shows that as the ANC decreases, the risk of developing oral ulcers increases. In accordance with a previous study conducted by Williams & Martin in 1992 which discovered a statistically significant association between neutropenia and the occurrence of oral ulcers in all acute leukemia patients undergoing chemotherapy and that ANC was especially low when oral ulcers were present. However, the study did not include a comparison of patients receiving chemotherapy without developing neutropenia.^[22]^ Similarly, Jena et al in 2022 reported a rate of 4.35% for oral ulcers in a group of cancer patients undergoing chemotherapy.^[10]^ Other studies, such as those by Chen et al in 2004 and Santilal & Graça in 2019, have reported higher rates of ulcers due to chemotherapy, ranging from 40% to 30.4%.^[19,23]^ In contrast, Subramaniam et al in 2008 reported a slightly higher rate of 5.2% of oral ulcers,^[17]^ and Barret in 1987 found that 9.09% of ulcer cases were directly caused by chemotherapy.^[24]^

Moreover, patients diagnosed with CIN are prone to a range of bacterial, fungal, and viral infections, particularly those affecting the oral cavity, and these infections often have a tendency to recur.^[25]^ It has been observed that Candida albicans, a fungus commonly found in oral flora, can lead to opportunistic fungal infections in neutropenic patients. The presence of neutrophils is vital in defending against pathogens, including fungal infections.^[3,11]^ However, a study by Okunaka et al in 2021 reported no occurrences of fungal infections in 85% to 92% of patients exhibiting grade III neutropenia.^[26]^ Similarly, in a study conducted by Muhammad & Alzubaidee in 2020, a statistically significant association between chemotherapy and fungal infections, suggesting a potential link with neutropenia.^[11]^ Several studies have investigated the frequency of fungal infections in patients with CIN. For instance, Williams & Martin in 1992 reported a higher rate of CIN-associated fungal infections among 50% of patients receiving chemotherapy.^[22]^ In contrast, Subramaniam et al in 2008, documented a lower rate of fungal infections, occurring in only 2 out of 58 patients diagnosed with acute lymphocytic leukemia who were receiving chemotherapy.^[17]^ Despite these varying outcomes, it is important to note that neutropenia can still contribute to fungal infections. In our study, the incidence of fungal infections in the CIN group was comparatively low. This may be attributed to the utilization of prophylactic antifungal rinses as part of patient management protocol. It is worth mentioning that patients who experienced fungal infections were more likely to exhibit severe myelosuppression, along with grade III and IV neutropenia. Furthermore, in the chemotherapy group, the occurrence of fungal infections was low due to the absence of myelosuppression and immunosuppression, these patients also administered prophylactic antifungal rinses as part of patient management protocol. As another example, other studies conducted by El-Housseiny et al in 2007 and Jena et al in 2022, reported higher rates of fungal infections (30% and 18.84%, respectively).^[10,27]^ On the other hand, studies conducted by Santilal & Graça in 2019 and Ponce-Torres et al in 2010 found lower rates of fungal infections (10.2% and 6.12%, respectively).^[23,28]^ These variations in rates may be influenced by the different population groups, treatment regimens, and precautionary measures used.

Myelosuppressed patients are susceptible to herpes lesions resulting from reactivation of the herpes simplex virus. These lesions are often characterized by pain and can be associated with bleeding, necrosis, as well as secondary bacterial and fungal infections.^[14,29,30]^ Similarly, indirect oral side effects of chemotherapy primary arise from neutropenia and impaired salivary enzymes, which increase the vulnerability of the patient to viral infections. This claim is supported by studies conducted by Muhammad & Alzubaidee in 2020 and Velten in 2017.^[11,23]^ However, differences in findings between this study and previous studies can be noted. For example, Ramírez-Amador et al in 1996 demonstrated an association between viral infection and neutropenia, reporting a viral infection rate of 28% in patients with hematological malignancies undergoing chemotherapy.^[31]^ On the other hand, Jena et al in 2022 and Muhammad & Alzubaidee in 2020 reported lower rates of viral infections (2.17% and 10%, respectively) in patients undergoing chemotherapy, potentially due to reactivation of latent viruses during immunosuppression.^[10,11]^ The decline in viral infection rate observed in neutropenic patients in our study may be attributed to the majority of participants having no current or past exposure to herpes simplex virus, thereby reducing the virus ability to reactivate during neutropenia. In the chemotherapy group, the low rate of viral infections can be attributed to the absence of myelosuppression, immunosuppression, as well as previous or current viral infections. However, Santilal & Graça in 2019 reported a lower viral infection rate observed in only 1 patient (2.2%).^[23]^ In contrast, Ponce-Torres et al in 2010 and Muhammad & Alzubaidee in 2007 found higher prevalence rates of viral infections (12.24% and 10.7%, respectively).^[11,28]^ These variations in viral infection rates can be attributed to differences in study populations, treatment regimens, and history of viral exposure.

Stomatitis, also known as Oral Mucositis, is a common complication in patients with malignancy who develop CIN. It is characterized by redness and extensive ulcers in the oral mucosa. The occurrence of mucositis in these patients may be due to many factors.^[14,32,33]^ Toxic chemotherapeutic agents contribute to mucositis through various mechanisms. Firstly, mucositis can occur as a direct consequence of the effects of chemotherapy on rapidly regenerating oral tissues. These drugs may alter the natural barrier function of the oral mucosa, leading to an increase in inflammatory reactions and mucositis.^[33–35]^ Secondly, chemotherapeutic agents cause inhibition in the defensive mechanisms mainly due to neutropenia, making the oral mucosa more susceptible to invasion by gram-negative bacteria and fungi. Additionally, there appears to be a transient association between ulcerative stomatitis and the occurrence of neutropenia.^[14,32,33,35]^ Although no studies exist that specifically examine the association between neutropenia and oral mucositis; however, Williams & Martin in 1992 demonstrated erythematous changes in the oral cavity in 10 patients, ranging from mucositis to the presence of an erythematous halo around the ulcers.^[22]^ Findings from our study differ from those conducted by Muhammad & Alzubaidee in 2020 which revealed a statistically significant association between chemotherapy and oral mucositis with an incidence rate of 46.3%.^[11]^ Our results also contradict those presented by El-Housseiny et al in 2007 which showed a statistically significant association between chemotherapy and oral mucositis, with incidence rates of 53.3%.^[27]^ There were significantly more cases of oral mucositis reported in the study by Ponce-Torres et al in 2010, accounting for 38.77% of patients receiving chemotherapy.^[28]^ Other studies, such as those conducted by Subramaniam et al in 2008 and Santilal & Graça in 2019, reported the presence of oral mucositis in 20.6% and 18.4% of patients, respectively.^[17,23]^ However, Jena et al in 2022 reported a slightly lower incidence of chemotherapy-induced oral mucositis, with a rate of 10.14%.^[10]^ Differences in the reported incidence of oral mucositis can be attributed to the specific drugs used to treat malignancy, the drug particular side effects, and the drug origin. These factors may influence the severity and frequency of oral mucositis observed in patients undergoing chemotherapy.

It is crucial to prioritize preventing and managing possible complications related to cancer and its treatment, such as neutropenia, which can lead to serious and often fatal infections requiring hospitalization and antibiotic treatment. Such infections can delay or reduce the intensity chemotherapy treatment, potentially affecting the patient quality of life and survival.^[8,36,37]^ Furthermore, the oral cavity can be a major source of the short- and long-term complications associated with cancer treatment, as oral bacteria can cause bacteremia and sepsis during chemotherapy. As such, it is essential to carefully assess and rule out potential sources of oral infections before initiating cancer treatment Patients undergoing chemotherapy who have oral mucositis and neutropenia are at a 4-fold greater risk of developing sepsis due to oral mucosal damage and ulcerative lesions that act as gateways for the oral microbiota to enter.^[37,38]^ The wide variety of clinical manifestations presented by neutropenia, some of which can be life-threatening, means that oral manifestations are often the first observed. Therefore, dentists must investigate the underlying disease causing these symptoms.^[39]^

This study represents the first global cross-sectional investigation to examine the relationship between neutropenia, chemotherapy, and oral manifestations. It also provides a comparison of the effects of chemotherapy alone and chemotherapy-induced neutropenia on oral health. Notably, details about the specific chemotherapy types, doses, and cycle numbers for each patient were not provided in this study. These factors can potentially affect oral health outcomes. Also, it is important to acknowledge that the participants in this study were exclusively from Syria, limiting the generalizability of the findings to other populations. Therefore, further research is recommended to corroborate and expand upon these results in diverse populations. Additional studies should aim to validate the relationship between neutropenia, chemotherapy, and oral manifestations, while also investigating potential interventions or preventive measures to mitigate the impact of neutropenia on oral health.

## 5. Conclusion

The findings highlight the need for close monitoring and treatment of neutropenia to prevent specific oral lesions, as the frequency of oral ulcers increases with increasing severity of neutropenia. However, no significant increase was observed in other oral lesions among neutropenic patients.

## Acknowledgments

We are grateful for the department of Hematology-Oncology of the University Hospitals Al-Bairouni and Al-Assad and staff who made this study possible. we also thank all contributors and patients. We gratefully acknowledge the generous support provided by Stemosis for Scientific Research, from Stemosis we thank Dr Majd Hanna for her advisory role.

## Author contributions

**Conceptualization:** Fatima AlZahraa Al Beesh, Siham Suleiman, Abeer Aljoujou.

**Data curation:** Fatima AlZahraa Al Beesh.

**Investigation:** Fatima AlZahraa Al Beesh.

**Methodology:** Fatima AlZahraa Al Beesh, Siham Suleiman, Abeer Aljoujou.

**Supervision:** Siham Suleiman, Abeer Aljoujou.

**Validation:** Fatima AlZahraa Al Beesh, Siham Suleiman, Abeer Aljoujou.

**Visualization:** Fatima AlZahraa Al Beesh, Siham Suleiman, Abeer Aljoujou.

**Writing – original draft:** Fatima AlZahraa Al Beesh.

**Writing – review & editing:** Nafiza Martini, Siham Suleiman, Abeer Aljoujou.
